# Tubulin glutamylation: a key regulator of flagella, cilia, centrosomes, and disease pathways

**DOI:** 10.1186/s12929-026-01244-z

**Published:** 2026-05-04

**Authors:** Shiau-Chi Chen, Yi-Chien Chuang, Yu-Chun Lin

**Affiliations:** 1 Institute of Molecular Medicine, National Tsing Hua University, Hsinchu, Taiwan; 2 Department of Medical Science, National Tsing Hua University, Hsinchu, Taiwan

**Keywords:** Tubulin glutamylation, Post-translational modifications, Cilia, Flagella, Centrosome, Neuron, Ciliopathies, Neurodegeneration

## Abstract

Tubulin glutamylation is an essential post-translational modification that expands the functional diversity of microtubules in many cellular structures, including flagella, motile cilia, primary cilia, centrosomes, and neurons. This modification adds variable lengths of glutamate side chains to the C-terminal tails of tubulin, creating a finely tuned biochemical signal that regulates microtubule stability, motor protein movement, the activity of severing enzymes, and the recruitment of key signaling molecules. Growing evidence shows that glutamylation is not uniformly distributed but instead forms distinct spatial patterns along microtubule arrays, particularly within the axonemes of flagella and cilia, centriolar triplets, and long-lived neuronal microtubules. These patterns are established by tubulin ligase–like enzymes that add glutamates and by carboxypeptidases that remove them, together shaping a dynamic “tubulin code.” In motile cilia and flagella, glutamylation fine-tunes dynein-driven force generation and the coordination of axonemal bending. Disruption of this modification impairs ciliary beating and sperm flagellar motility, leading to disorders such as primary ciliary dyskinesia, which manifests as chronic respiratory infections and laterality defects, and can also disrupt cerebrospinal fluid flow, causing hydrocephalus and male infertility such as asthenozoospermia. In primary cilia, reduced glutamylation perturbs intraflagellar transport and ciliary signaling and contributes to ciliopathies including Joubert syndrome and retinal degeneration. In dividing cells, altered glutamylation on centrosomes leads to errors in chromosome segregation and is associated with cancer progression. This review summarizes current knowledge of the enzymes, structural principles, and cellular mechanisms governing tubulin glutamylation, highlights its emerging roles in human diseases, and discusses new technological advances—including biochemical reconstitution, super-resolution imaging, and live-cell manipulation tools—that are beginning to reveal how this modification dynamically controls microtubule properties and the functions of flagella, cilia, and centrosomes in health and disease.

## Introduction

**Fig. 1:**
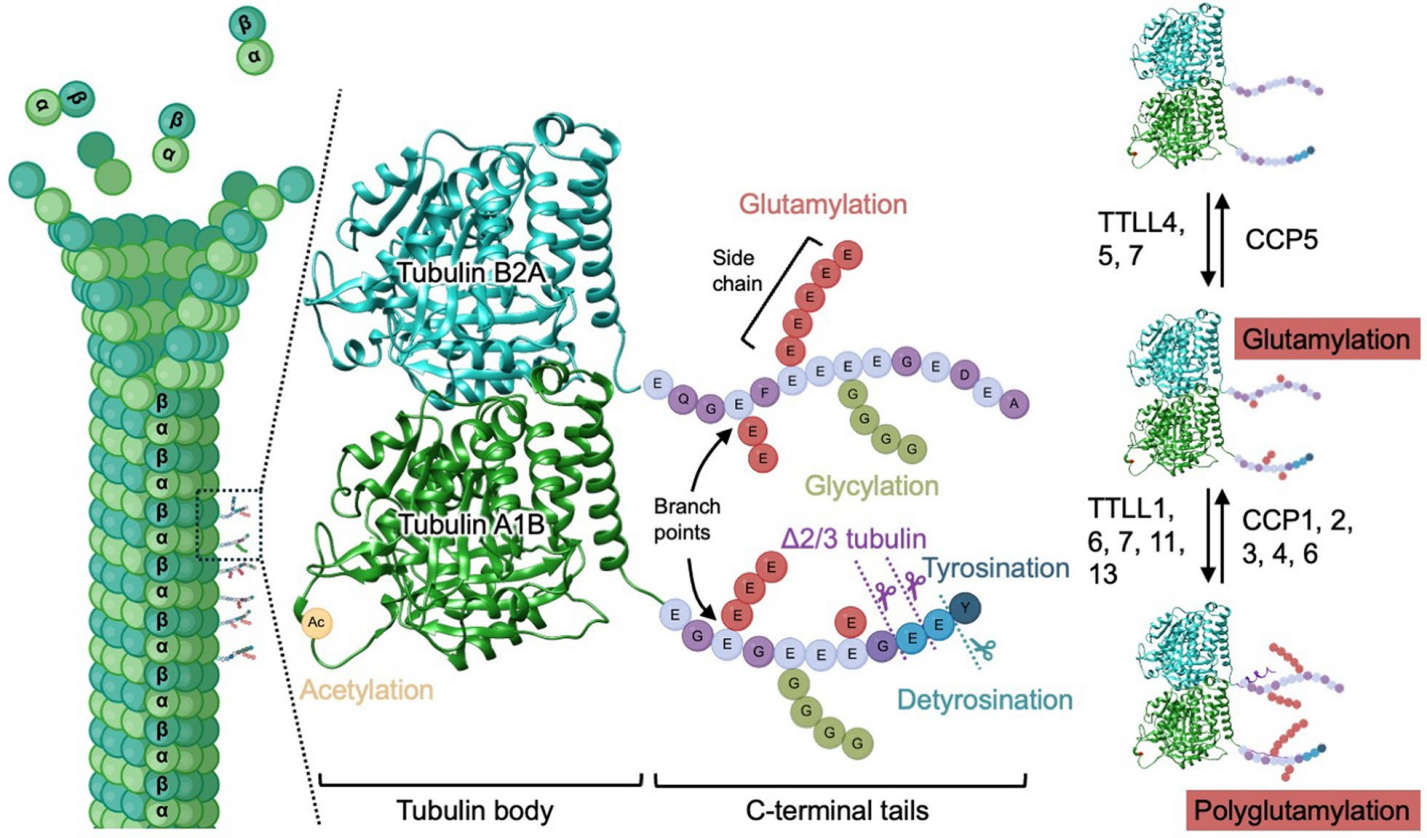
Tubulin C-terminal tail modifications and enzymes regulating glutamylation. Microtubules (MTs) consist of polarized α/β-tubulin heterodimers that assemble into cylindrical polymers. The structural model illustrates α-tubulin (green) and β-tubulin (cyan), with acetylation occurring on lysine residues located within the tubulin body. In contrast, the flexible C-terminal tails (CTTs) of both subunits serve as primary hotspots for diverse post-translational modifications, including glutamylation, glycylation, tyrosination/detyrosination, and the generation of Δ2/Δ3 tubulin from detyrosinated tubulin. Glutamylation entails the sequential addition of glutamate residues, forming side chains of variable length branching from specific glutamate sites. Glycylation introduces glycine residues onto the CTTs, whereas tyrosination and detyrosination regulate the reversible addition or removal of the terminal tyrosine. The right panel summarizes the enzymes that regulate tubulin glutamylation. TTLL family members initiate (e.g., TTLL4, TTLL5, TTLL7) or elongate (e.g., TTLL1, TTLL6, TTLL7, TTLL11, TTLL13) glutamate side chains, whereas CCP enzymes counteract this process by removing glutamate units. CCP5 uniquely cleaves the initiating branching glutamate, while CCP1, CCP2, CCP3, CCP4, and CCP6 trim elongating polyglutamate chains. Together, these PTMs and their regulatory enzymes orchestrate MT dynamics, stability, and interactions with MT-associated proteins. *Note* Structures of tubulin α1B and β2A were generated using AlphaFold. CTTs are not drawn to scale. The schematic illustration was partially created using BioRender.

Tubulin is a highly conserved cytoskeletal protein that serves as the fundamental building block of microtubules (MTs), which are essential for various cellular processes such as maintaining cell shape, intracellular transport, cell migration, and cell division [1]. The basic unit of tubulin is a heterodimer composed of two globular subunits, α-tubulin and β-tubulin, each approximately 450 amino acids long. Extending from the globular body of each subunit is an intrinsically disordered C-terminal tail (CTT) (Fig. 1). While the globular body is highly conserved across species, the CTTs are more diverse and protrude from the surface of assembled MTs. These tails are critical functional hotspots, serving as the primary sites for post-translational modifications (PTMs) and as interaction hubs for MT-associated proteins (MAPs) and molecular motors such as kinesins and dyneins [2]. Tubulin heterodimers polymerize in a head-to-tail manner into protofilaments, which then associate laterally to form the hollow, cylindrical MTs (Fig. 1). While a 13-protofilament structure is common in many organisms, this number can vary considerably, giving rise to specialized MT architectures across different cell types and functional contexts [1].

The functional diversity of MTs is regulated by a complex system known as the "tubulin code" [1, 2]. This code arises from two primary sources: the incorporation of different tubulin isotypes into the MT lattice and the addition of various PTMs [3–19] (Fig. 1). Most organisms express multiple genes for both α- and β-tubulin; for instance, the human genome encodes more than nine of each [20], whereas *C. elegans* has nine α- and six β-tubulins. This allows cells to create specialized MT networks by using a distinct "palette" of tubulin isoforms. The diversity is further amplified by numerous PTMs, which are covalent chemical changes that often occur on the CTTs of tubulins after they have been incorporated into MT polymers [1].

Key PTMs that contribute to the tubulin code include glutamylation, the addition of glutamate chains to tubulin tails [3]; glycylation, the addition of glycine chains [4]; acetylation of Lysine 40 on α-tubulin within the MT lumen [5–7]; and the detyrosination/tyrosination cycle at the CTT of α-tubulin [8, 9]; and irreversible C-terminally truncated forms of α-tubulin, ∆2- and ∆3-tubulin, generated by the sequential removal of C-terminal amino acids [10–12] (Fig. 1). In addition to these well-studied modifications, tubulin also undergoes polyamination [13], palmitoylation [14, 15], and phosphorylation [16], which are less understood but may further diversify the regulatory potential of the tubulin code. Rare modifications such as ubiquitination [17, 18] and SUMOylation [19] have also been reported, suggesting an even broader landscape of regulatory inputs. These PTMs act as biochemical signals that are "read" by other cellular proteins, thereby regulating MT stability, dynamics, and interactions with effectors [1, 2].

Among tubulin PTMs, glutamylation is a complex and critical PTM that profoundly affects neuronal health [21]. Unlike simple binary PTMs, it functions as a “rheostat,” modulating MT behavior; dysregulation of glutamylation leads to ciliopathies and neurodegeneration, highlighting its central role in the tubulin code [22]. Here, we focus on glutamylation and comprehensively describe the approaches used to study tubulin glutamylation and highlight its roles in different physiological and pathological conditions.

### Tubulin glutamylation and its enzymatic machinery

Tubulin glutamylation is a reversible PTM that fine-tunes MT functions, and its dynamic nature is governed by the coordinated actions of two opposing enzyme families: the “writers” and the “erasers.” The writers are the tubulin tyrosine ligase-like (TTLL) glutamylases [23], which add glutamate residues to the CTTs of α- and β-tubulin, whereas the erasers are the cytosolic carboxypeptidases (CCPs), which remove these side chains [24, 25]. The balance between these two activities determines the length, position, and frequency of glutamate side chains, ultimately shaping the “tubulin code” that regulates MT stability, motor protein behavior, and effector recruitment in a context-dependent manner [22]. Structurally, TTLL enzymes share a conserved ATP-grasp ligase fold homologous to tubulin tyrosine ligase, with an ATP-binding motif essential for glutamate ligation. Many TTLLs also contain a flexible, positively charged MT-binding domain (c-MTBD) that enhances lattice interaction, enabling efficient addition of glutamate side chains onto MTs [26, 27]. The TTLL family includes nine enzymes (TTLL1, -2, -4, -5, -6, -7, -9, -11, and -13) that either initiate or elongate glutamate side chains with distinct substrate preferences and functional roles [26, 28–30] (Fig. 1). TTLL4 and 5 primarily catalyze the installation of the initial branch-point glutamate, whereas TTLL1, -6, -9, -11, and -13 predominantly mediate chain elongation; TTLL7 is unusual in that it can support both initiation and elongation reactions [23, 29–32] (Fig. 1). Regarding substrate preference, TTLL1, -5, -6, -9, -11, and -13 preferentially modify α-tubulin, whereas TTLL4 and TTLL7 display a bias toward β-tubulin [23, 28–32]. Structural and biophysical studies have revealed the molecular basis of these preferences. For example, analysis of TTLL7 uncovered a dedicated MT-interaction module that establishes polymer-specific contacts with the β-tubulin CTTs, thereby explaining its protomer specificity [26]. In contrast, TTLL1 lacks an autonomous MT-binding domain and functions within a multisubunit complex, highlighting the mechanistic diversity within the TTLL family [26]. Moreover, a combination of structure-guided inhibitor and mutational analyses demonstrated that elongation activity, exemplified by TTLL6, is encoded by distinct active-site features that favor α-linked glutamate chain propagation [29]. Collectively, these findings indicate that both initiation versus elongation and α- versus β-tubulin specificity are determined by discrete structural elements rather than overall sequence homology, enabling the generation of diverse glutamylation patterns on MTs. Notably, elongation activity is further modulated by the modification state of the MT substrate itself. TTLL6 displays a markedly enhanced catalytic efficiency toward detyrosinated α1A/βIII MTs, exhibiting an approximately 20-fold increase in activity compared with tyrosinated MTs, suggesting that in vivo TTLL6 may preferentially extend glutamate side chains on detyrosinated α-tubulin [29].

In contrast, CCP enzymes counteract TTLL activity by trimming or completely removing glutamate side chains from tubulin [24, 25]. They are zinc-dependent exopeptidases that exhibit distinct substrate specificities: CCP1, 2, 3, 4, and 6 preferentially shorten α-linked glutamate side chains, while CCP5 uniquely removes the branching γ-linked glutamate that initiates chain formation [24, 25, 33, 34] (Fig. 1). Importantly, TTLLs act mainly on polymerized MTs, whereas CCPs preferentially target soluble tubulin dimers, enabling cyclical resetting of glutamylation states during MT turnover [35]. This spatial division ensures that chain elongation occurs on stable MTs, whereas trimming and complete removal occur upon tubulin recycling.

Together, TTLL writers and CCP erasers form a tightly coordinated enzymatic network that writes, edits, and erases glutamate side chains in a precise manner. Through structural specialization, substrate selectivity, temporal control, and spatial regulation, these enzymes generate combinatorial patterns that constitute the tubulin glutamylation code, allowing cells to adapt MT behavior to diverse physiological contexts and prevent dysfunction associated with human diseases.

### Visualization and characterization of tubulin glutamylation

**Fig. 2:**
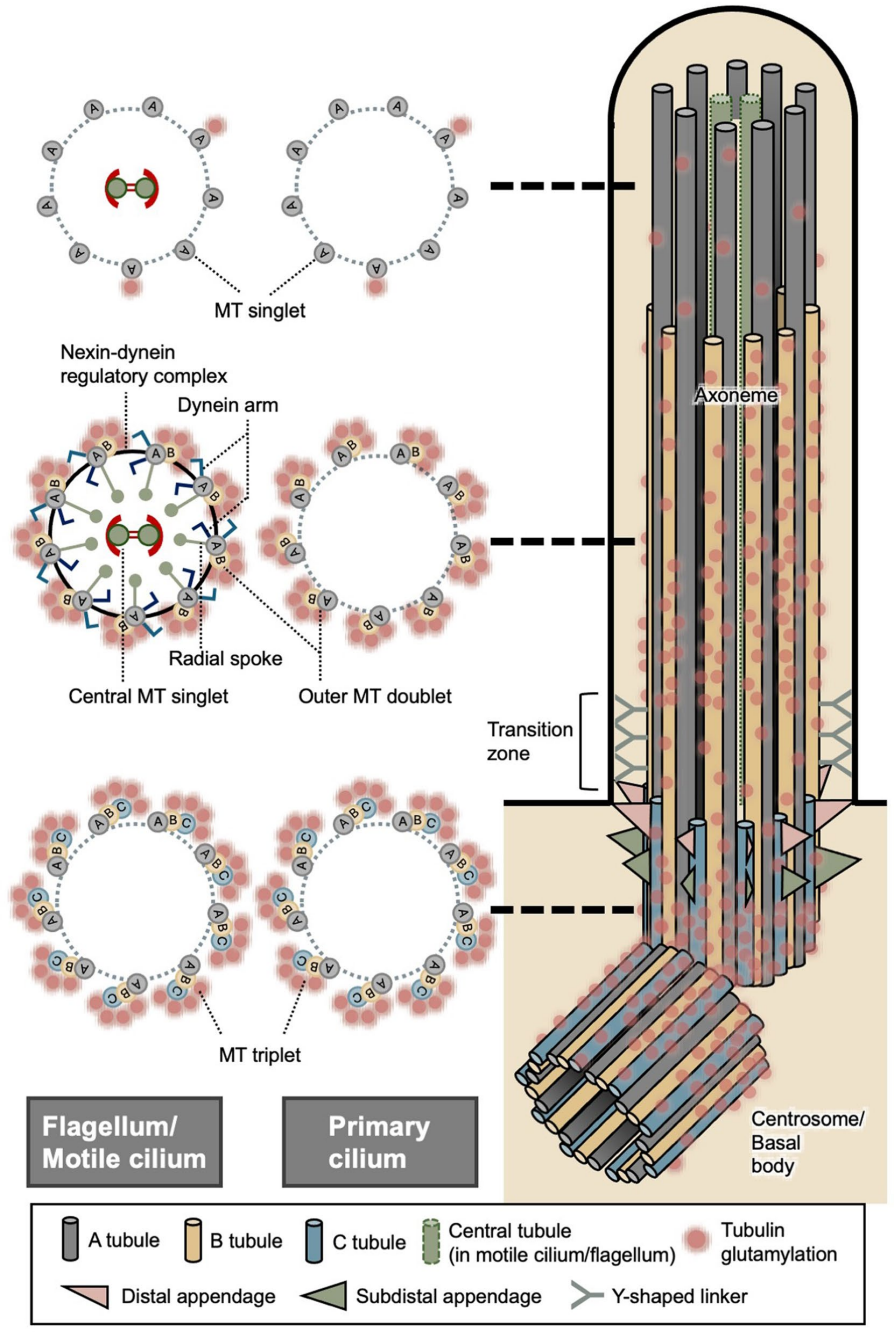
Compartment-specific distribution of tubulin glutamylation in flagella/motile cilia, primary cilia, and centrosomes. The schematic illustrates the microtubule architecture of the centrosome/basal body and flagellar/ciliary axoneme together with the spatial distribution of tubulin glutamylation. The longitudinal view on the right shows the overall organization of the centrosome/basal body, transition zone, and axoneme, whereas the circular diagrams on the left represent cross-sectional views corresponding to specific positions along this structure. Motile cilia and flagella possess a canonical 9 + 2 axonemal structure composed of nine outer doublet microtubules surrounding a central pair, whereas primary cilia typically exhibit a 9 + 0 configuration lacking the central pair. In primary cilia, the proximal and middle axoneme contain microtubule doublets, while in the distal region the B-tubules terminate, producing a doublet-to-singlet transition. Tubulin glutamylation (red dots) shows distinct compartment-specific patterns. In axonemes, glutamylation is enriched on B-tubules of doublet microtubules and generally decreases toward the distal region. At the centrosome and basal body, glutamylation is abundant on centriolar triplet microtubules

Tubulin glutamylation is commonly analyzed using antibody-based methods and mass spectrometry. Immunofluorescence and western blotting, together with well-characterized antibodies such as GT335 (glutamylation), PolyE, B3, and 1D5 (poly- or long-chain glutamylation), enable visualization and semi-quantitative analysis of different modification states in cells and tissues [3, 36–39]. These antibodies have revealed that glutamylation is not uniformly distributed but instead enriched on stable MTs in specific subcellular structures such as ciliary axonemes, sensory cilia in *C. elegans*, and mammalian photoreceptors [30, 37, 40]. Notably, strong glutamylation signals are also consistently detected along the axonemal MTs of flagella and motile cilia [36, 41, 42]. In the nervous system, glutamylation is abundant in the brain and increases during postnatal maturation, with particularly high enrichment in Purkinje cells [21]. High levels are also consistently detected at MT-organizing centers such as centrioles and basal bodies [37, 38, 43] (Fig. 2), and during mitosis, glutamylation appears on spindle MTs and the midbody [44].

For molecular characterization, mass spectrometry (MS) is the gold standard. Liquid Chromatograph-Tandem MS identifies specific glutamylation sites, while intact protein MS measures chain length. MS established that α-tubulin can carry 3–6 glutamate residues and up to ~ 11 residues in brain tissue [45–47]. Enzyme profiling showed that TTLL4 initiates glutamate addition and TTLL6/TTLL7 elongate chains with distinct preferences for α- or β-tubulin [23, 30, 44]. Deglutamylases such as CCP1 were validated by MS to shorten chains [25, 47]. Importantly, MS-based studies have also revealed crosstalk within the tubulin code, demonstrating that polyglutamylation enhances detyrosination, and β-tubulin glutamylation can recruit TTLL6 to α-tubulin, thereby creating a positive feedback regulatory mechanism [29, 48]. Together, these studies establish MS as an indispensable tool for understanding the molecular logic of tubulin glutamylation and the broader “tubulin code” [1, 2].

### Spatial distribution of tubulin glutamylation in flagella/motile cilia

Motile cilia and flagella are structurally homologous organelles that share a highly conserved axonemal MT architecture. Both are built around an axoneme typically organized in a canonical 9 + 2 arrangement, consisting of nine peripheral outer doublet MTs surrounding a central pair of singlet MTs, together with associated dynein arms, radial spokes, and nexin–dynein regulatory complexes [49, 50] (Fig. 2). Coordinated ATP-dependent movement of axonemal dynein motors along adjacent outer doublets generates inter-doublet sliding, which is mechanically constrained by cross-linking structures and converted into bending of the axoneme, thereby producing rhythmic beating and oscillatory motion. While motile cilia are commonly found on the surface of epithelial cells in multicellular organisms, flagella are typically present in sperm cells and in many unicellular eukaryotes.

Axonemes in the flagella of several protists are highly enriched in tubulin glutamylation, making these organisms particularly suitable model systems for investigating glutamylation. In both *Tetrahymena* and *Chlamydomonas*, glutamylated tubulin is predominantly localized to the axonemal outer doublet MTs, whereas the central pair MTs exhibit little or no detectable modification [51, 52] (Fig. 2). At higher spatial resolution, glutamylation within the outer doublets of flagella is preferentially enriched on the B-tubule rather than the A-tubule [51–53] (Fig. 2). Moreover, glutamylation displays a longitudinal gradient along the axoneme, with higher levels near the proximal region that progressively decrease toward the distal tip [51] (Fig. 2). In contrast to protist flagella, where glutamylation is largely concentrated on the outer doublet MTs, *Drosophila* sperm display extensive long-chain polyglutamylation predominantly on the central pair and accessory MTs, whereas the outer doublets contain only small amounts of glutamylated tubulin [54].

### Spatial distribution of tubulin glutamylation in primary cilia

In contrast to motile cilia and flagella, which generate active beating through dynein-driven MT sliding, primary cilia are generally immotile sensory organelles. Most primary cilia display a 9 + 0 axonemal architecture, lacking the central pair and dynein arms required for motility (Fig. 2). Rather than producing mechanical force, primary cilia function as specialized signaling hubs that detect extracellular chemical and mechanical cues and transduce them into intracellular responses [55].

The structural organization of the primary cilium, including the transition zone and the axoneme (Fig. 2), establishes a compartmentalized platform that regulates selective protein entry and spatially restricted signaling events [56–59]. Tubulin glutamylation in primary cilia exhibits a distinct regional and longitudinal distribution along the axoneme. The axonemal MTs within the transition zone display relatively low levels of glutamylation. Beyond the transition zone, glutamylation is highest in the proximal region of the axoneme and progressively decreases toward the distal region of the primary cilium [43, 60–62] (Fig. 2). However, the direct ultrastructural evidence clarifying whether glutamylation preferentially localizes to the A- or B-tubule within primary ciliary doublet MTs remains limited. A recent study employing expansion microscopy to examine the photoreceptor connecting cilium revealed that tubulin glutamylation and long-chain polyglutamylation are predominantly concentrated on the outer doublet MTs [62]. High levels of glutamylation were detected in the middle segment of the axoneme, where both A- and B-tubules are present, whereas the distal segment—composed predominantly of A-tubules—showed little or no detectable glutamylation. Importantly, loss of tubulin glutamylation was associated with structural defects in the B-tubule [62–64] (more details in later sections). Taken together, these observations suggest that, although direct tubule-specific mapping is still lacking, tubulin glutamylation in primary cilia is likely preferentially associated with the B-tubule and contributes to the structural stability of axonemal doublet MTs.

### Spatial distribution of tubulin glutamylation in centrosomes/basal bodies

The centrosome serves as the primary MT-organizing center in most animal cells and fundamentally depends on tubulin to build its architecture, nucleate MTs, and regulate diverse cellular processes [65–68]. At its core, the centrosome contains a pair of centrioles arranged orthogonally, each formed by a specialized cylindrical scaffold of nine MT triplets built from α/β-tubulin [69, 70]. Within each triplet, the A-tubule is a complete 13-protofilament MT that provides the structural foundation, while the B- and C-tubules are incomplete MTs sharing protofilaments with their neighboring tubules (Fig. 2), creating a rigid and stable framework essential for centriole and basal body integrity [71]. This triplet-based structure distinguishes centriolar MTs from the highly dynamic cytoplasmic MTs and ensures long-term stability.

Surrounding the centrioles is the pericentriolar material (PCM), which is enriched in γ-tubulin ring complexes that template and anchor cytoplasmic MTs, thereby positioning the centrosome as the dominant organizer of the MT cytoskeleton [65, 72]. Through the polymerization of α/β-tubulin to form the centriolar scaffold and the use of γ-tubulin for MT nucleation, tubulin enables the centrosome to assemble bipolar mitotic spindles, organize interphase MT arrays, and establish cell polarity.

Importantly, the functional identity of a centriole is context dependent. In cycling cells, centrioles act as core components of the centrosome, whereas upon cell cycle exit or entry into quiescence, the mature mother centriole is converted into a basal body that nucleates and anchors the primary cilium (Fig. 2)**.** This conversion involves the docking of the mother centriole to the plasma membrane via its distal appendages [67], followed by the extension of axonemal MTs from the basal body template. During the cell cycle, centrosomes duplicate once in S phase and separate in mitosis to form two spindle poles, where tightly regulated MT nucleation ensures accurate chromosome segregation [73]. Beyond its structural role, the centrosome functions as a signaling hub, integrating tubulin-based scaffolding with pathways that regulate cell cycle progression, polarity determination, and developmental patterning [66].

Glutamylation is highly enriched on centriolar MTs and displays striking spatial and structural specificity, as revealed by super-resolution imaging approaches. In *Chlamydomonas*, structured illumination microscopy (SIM) and ultrastructure expansion microscopy analyses using GT335 and PolyE antibodies have shown that glutamylation is confined to the external surface of MT triplets, precisely where the PCM is located [74, 75] (Fig. 2). Collectively, these observations indicate that centriolar glutamylation follows a highly defined spatial pattern along both the longitudinal and radial axes, suggesting a critical role in maintaining centriole organization and mechanical stability.

### Localization, trafficking, and regulation of glutamylation modifying enzymes

TTLL4, TTLL5, TTLL6, and TTLL7 have been detected at both basal bodies and cilia, whereas TTLL1, TTLL9, and TTLL11 appear restricted to basal bodies [30]. In contrast, the deglutamylase CCP5 was reported to distribute uniformly along the entire axonemal shaft [60, 76]. Because the spatial balance between glutamylating and deglutamylating enzymes determines the tubulin glutamylation landscape, their precise subcellular localization is likely a key determinant of region-specific glutamylation patterns. The ciliary entry of glutamylases such as TTLL6 is regulated by specific targeting mechanisms. Vesicle trafficking pathways control their trafficking to and import into cilia. For example, the ARL13B–RAB11–FIP5 axis promotes the delivery of TTLL5/6-containing vesicles to the ciliary base, facilitating TTLL6 entry and axonemal polyglutamylation [60]. In addition, the basal body/transition zone protein CEP41 is required for TTLL6 entry into primary cilia; mutations in CEP41 disrupt TTLL6 localization and impair axonemal tubulin glutamylation, highlighting the importance of specific docking factors at the ciliary gate for TTLL6 targeting [64]. Cell cycle–associated kinases can further influence this process: CDK7–CDK6–mediated phosphorylation of FIP5 disrupts its interaction with RAB11, reducing TTLL6 import and leading to hypoglutamylation of axonemal MTs [77]. Beyond regulating ciliary entry, glutamylase activity itself can also be modulated. In sensory cilia of *C. elegans*, p38 MAPK–dependent phosphorylation of TTLL-4 at Thr446 enhances its catalytic activity, thereby increasing ciliary tubulin glutamylation [40]. Together, these findings demonstrate that upstream signaling pathways can regulate both the localization and catalytic activity of glutamylation enzymes, thereby dynamically controlling the spatial distribution of tubulin glutamylation within cells.

### Approaches to manipulate tubulin glutamylation

Manipulating tubulin glutamylation can be achieved through genetic modulation of the enzymes that write or erase glutamate chains, biochemical reconstitution of the modification in vitro, and spatiotemporally controlled recruitment of enzymes in living cells. Overexpression of TTLL5 or TTLL7 in HeLa cells markedly increases glutamylation [78], whereas overexpression of TTLL6 or TTLL11 generates long glutamate side chains on tubulin [30]. Conversely, deletion of ttll-11 nearly abolishes glutamylation in *C. elegans* [79], whereas knockdown of ttll6 in zebrafish reduces ciliary glutamylation [80]. Similarly, knockout of Ttll1 in mice eliminates most glutamylated tubulin in tracheal cilia [42], and knockout of Ttll9 reduces polyglutamylation in sperm flagella [81]. On the other hand, loss of CCP activity leads to hyperglutamylation: mutation of ccpp-1 or ccpp-6 in *C. elegans* elevates ciliary glutamylation [63], whereas CCP5 knockdown increases glutamylation in zebrafish [82]. In contrast, overexpression of CCP enzymes reduces glutamylation, as shown by ccpp-6 overexpression in *C. elegans* and CCP5 overexpression in mammalian cells [24, 25, 34].

To directly assess the direct biochemical effects of glutamylation, researchers have reconstituted the modification process in vitro using purified tubulin and recombinant enzymes [83]. Unmodified tubulin from sources such as tsA201 cells provides a clean substrate, whereas brain tubulin, which is naturally rich in glutamylation, can be used to study deglutamylase activity or further chain elongation [83, 84]. Incubation of TTLL4, TTLL6, or TTLL7 with MTs in the presence of ATP and glutamate allows generation of defined glutamylation patterns depending on the enzyme, time, and tubulin isoform. For instance, TTLL6 adds long chains to α-tubulin, while TTLL7 modifies β-tubulin [29]. Conversely, recombinant CCP1 or CCP5 can be applied to glutamylated tubulin to shorten or fully remove modifications, and these enzymes act preferentially on soluble dimers and can function synergistically to reset glutamylation states [25, 35].

**Fig. 3:**
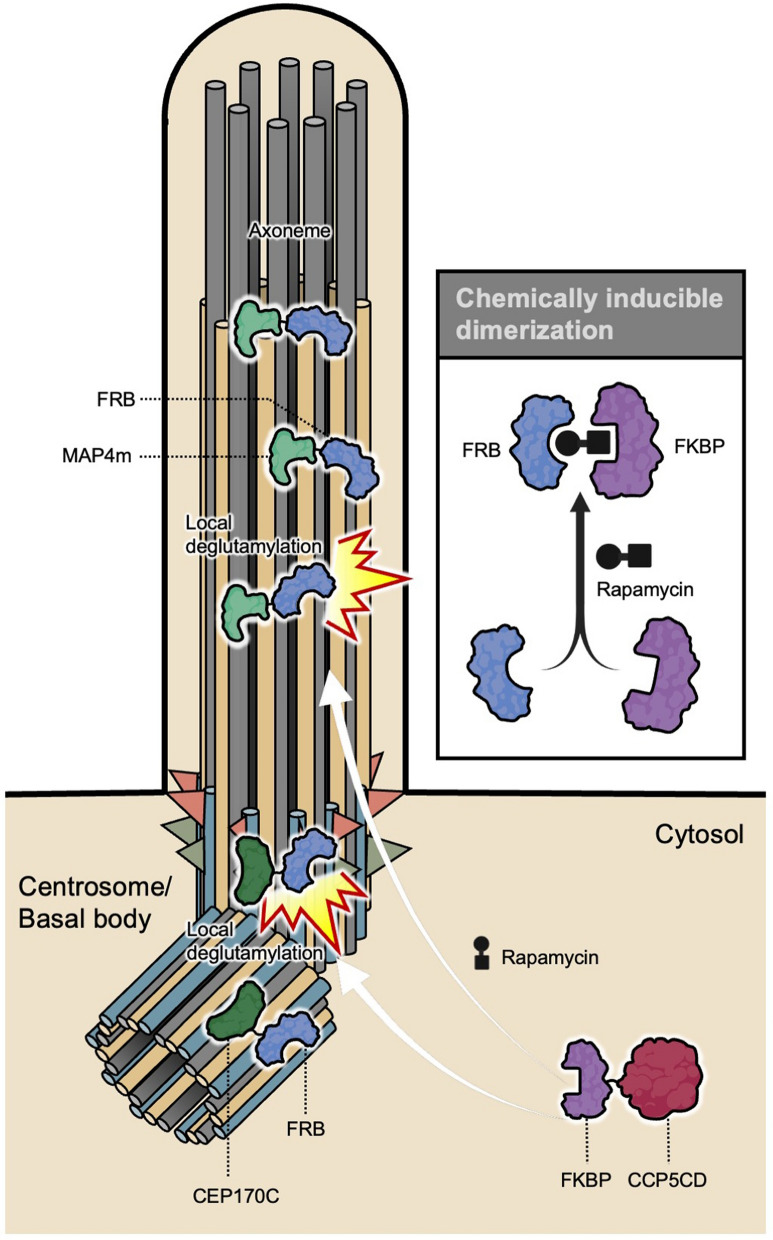
Chemically inducible recruitment of a deglutamylase to specific ciliary compartments for local depletion of tubulin glutamylation. Schematic illustration of a chemically inducible dimerization system used to locally manipulate tubulin glutamylation in target sites. A targeting module fused to FRB directs the system to specific compartments: CEP170C–FRB targets the centrosome and basal body, whereas MAP4m–FRB localizes along the ciliary axoneme. The deglutamylase module consists of the catalytic domain of CCP5 fused to FKBP (FKBP–CCP5CD) and is initially distributed in the cytosol. Upon addition of rapamycin, FKBP and FRB undergo rapid heterodimerization, recruiting CCP5CD to the targeted compartment. This recruitment induces local deglutamylation of microtubules at the centrosome/basal body or along the ciliary axoneme, enabling local and acute manipulation of tubulin glutamylation. The schematic illustration was partially created using BioRender

A breakthrough in manipulating glutamylation with high spatial and temporal precision in living cells is the STRIP (SpatioTemporal Rewriting of Intraciliary PTMs) system [22, 61, 85]. This approach uses chemically inducible dimerization (e.g., rapamycin-induced FKBP–FRB binding) to rapidly recruit catalytic domains of modifying enzymes to specific MT populations [61, 72, 85–87]. For example, an FRB–MAP4m (minimal fragment of MT-associated protein 4) fusion anchors to the ciliary axoneme, whereas the FKBP-fused catalytic domain of CCP5 (CCP5CD) remains cytosolic until rapamycin is added. Rapamycin triggers rapid recruitment of CCP5CD to the axoneme, leading to local and acute removal of glutamate side chains within minutes [61] (Fig. 3). This approach enables researchers to study immediate effects on processes such as intraflagellar transport (IFT) or Hedgehog signaling without long-term compensation. A similar strategy was later applied to centrosomes to assess the role of glutamylation there [43] (Fig. 3). Together, these genetic, biochemical, and optochemical strategies provide powerful and complementary approaches to modulate tubulin glutamylation with organismal, molecular, or subcellular precision.

### Intrinsic and extrinsic mechanisms of how glutamylation regulates MTs

**Fig. 4:**
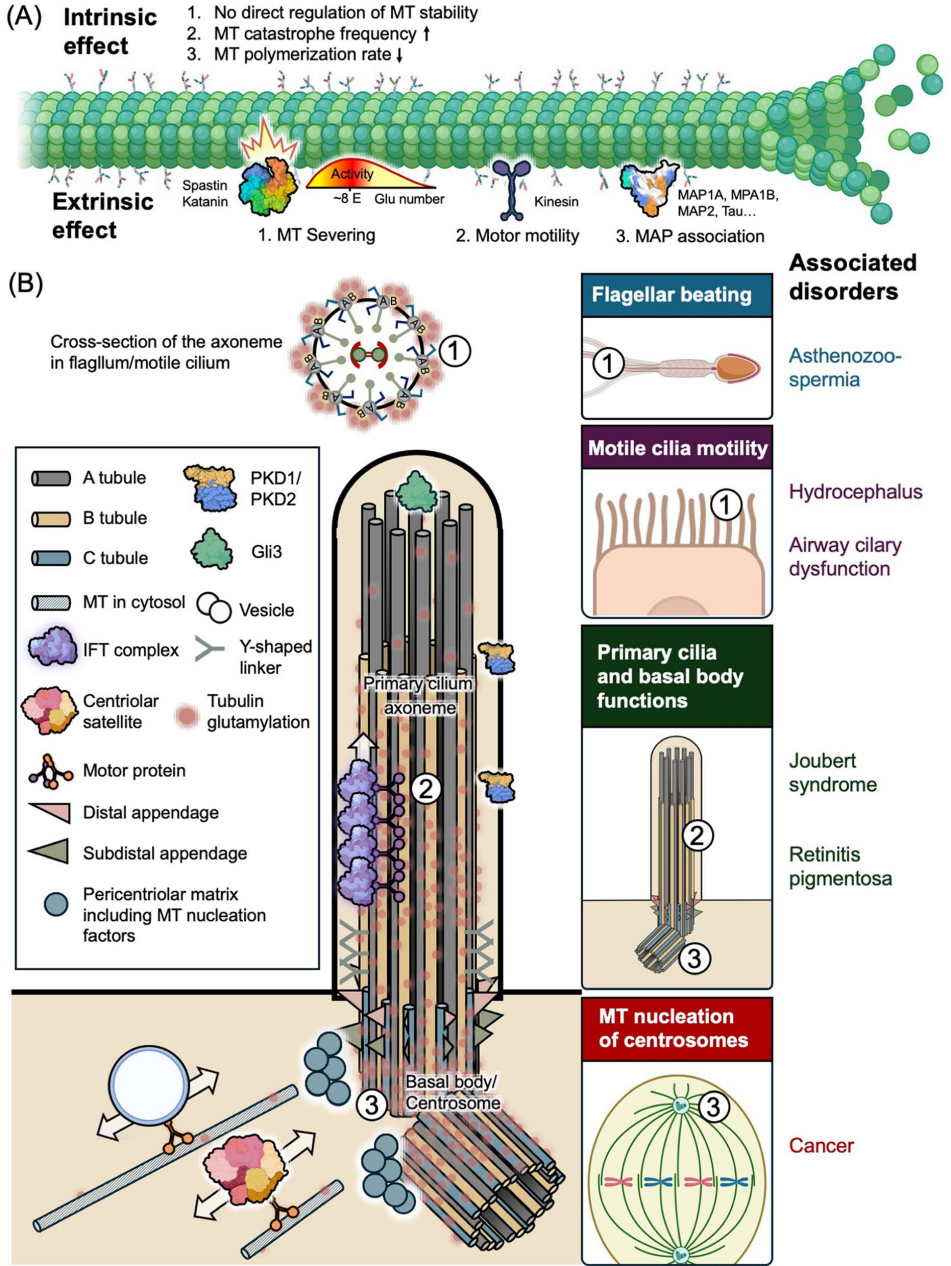
The roles of tubulin glutamylation in microtubules, flagella, motile cilia, primary cilia, and centrosomes. **A** Tubulin glutamylation modulates the intrinsic properties of microtubules and regulates their interactions with microtubule-associated proteins, motor proteins, and microtubule-severing enzymes. By altering the length and density of glutamate side chains on the C-terminal tails of tubulin, glutamylation influences motor activity, microtubule stability, and susceptibility to severing, thereby shaping microtubule organization and dynamics. **B** (1) In motile cilia and flagella, glutamylation on axonemal microtubules regulates dynein-driven sliding between outer doublets, thereby controlling flagellar beating and motile cilia motility. Disruption of this regulation can lead to impaired sperm motility (asthenozoospermia) and motile cilia–related disorders such as hydrocephalus. (2) In primary cilia, glutamylation along the axoneme regulates IFT and signaling. Perturbation of glutamylation disrupts primary cilium and basal body functions and is associated with ciliopathies, including Joubert syndrome, hydrocephalus, and retinitis pigmentosa. (3) At the centrosome, glutamylation of centriolar microtubules influences microtubule nucleation and organization, contributing to proper spindle assembly and chromosome segregation. Dysregulation of these processes has been linked to cancer. The schematic illustration was partially created using BioRender

Glutamylation is typically enriched on stable MT populations such as those in primary cilia, centrioles, and neuronal axons [3, 36–39]. A central question has been whether this modification intrinsically stabilizes MTs. Recent in vitro reconstitution studies have clarified this point. Chen and Roll-Mecak demonstrated that tubulin glutamylation acts as an intrinsic negative regulator of MT dynamics [35]. Using precisely modified tubulin species, they found that both glutamylation and polyglutamylation slow MT elongation rates and increase catastrophe frequency, without affecting GTP hydrolysis. Thus, the stability of glutamylated MTs observed in cells likely arises indirectly through the recruitment of MAPs, rather than the modification itself (Fig. 4A).

Beyond intrinsic effects on MT dynamics, glutamylation modulates the extrinsic interactions between MTs and their associated proteins, including severing enzymes, motor proteins, and signaling molecules [22]. Glutamylation critically tunes the activities of MT-severing enzymes such as Spastin and Katanin [78, 88, 89]. Spastin-mediated severing shows a biphasic response: activity increases with glutamate chain length up to approximately eight glutamate residues, but is inhibited when glutamate chains become excessively long [87]. Similarly, α-tubulin glutamylation strongly activates Katanin, whereas β-tubulin modification produces a dual effect—stimulatory at low glutamate chain lengths but inhibitory at high levels [89] (Fig. 4).

Glutamylation exerts diverse, context-dependent effects on kinesin motors. Long glutamate chains (~ 10 residues) can enhance the processivity of Kinesin-1 in vitro [90], whereas β-tubulin polyglutamylation catalyzed by TTLL7 has been shown to inhibit Kinesin-1 motility [91]. For Kinesin-2, glutamylation increases both processivity and velocity. For example, loss of CCPP-1 in *C. elegans* results in hyperglutamylation and accelerated movement of OSM-3/KIF17 [63]. Similarly, acute reduction of glutamylation in primary cilia perturbs motility of KIF3B and IFT [61] (Fig. 4). For Kinesin-3 motors, loss of α-tubulin polyglutamylation impairs KIF1A targeting [92], and imbalance between TTLL-11 and CCPP-1 disrupts KLP-6 motility in *C. elegans* cilia [93]. Collectively, these results demonstrate that the level and spatial pattern of glutamylation form a “tubulin code” that fine-tunes the behavior of MAPs and motors, thereby influencing MT organization and cellular function (Fig. 4).

### The roles of glutamylation in flagella/motile cilia

Direct genetic perturbations of glutamylation enzymes have established that tubulin glutamylation plays a regulatory rather than a structural role in flagellar and ciliary motility. In *Chlamydomonas reinhardtii*, genetic disruption of TTLL9 demonstrated that long-chain α-tubulin polyglutamylation is dispensable for axonemal assembly but essential for proper flagellar motility [53]. Loss of polyglutamylation selectively impaired inner dynein arm–driven force generation, leading to reduced flagellar beating. Interestingly, this defect paradoxically increased MT sliding under low-load conditions, suggesting that glutamylation fine-tunes dynein activity rather than serving a structural role in axoneme assembly [53]. Consistent with these findings, studies in *Tetrahymena* showed that TTLL6-mediated tubulin polyglutamylation is spatially enriched on the B-tubule of axonemal doublet MTs and is essential for normal ciliary motility [52]. Loss of TTLL6 increased inner dynein arm–driven MT sliding in the absence of outer dynein arms, demonstrating that tubulin glutamylation functions as a negative regulator of inner dynein arm activity and thereby fine-tunes ciliary waveform and beat frequency [52].

Genetic analyses in vertebrate systems further support a conserved role of tubulin glutamylation in regulating motile cilia mechanics. In zebrafish, knockdown of TTLL6 selectively reduced axonemal tubulin glutamylation, resulting in a pronounced decrease in ciliary beat amplitude while preserving overall ciliary structure [80]. Similarly, in mice, loss of TTLL9 led to reduced polyglutamylation in sperm flagella, accompanied by structural abnormalities in specific axonemal doublets and impaired flagellar beating [81]. These findings established tubulin glutamylation as a dominant PTM controlling ciliary motility, likely through modulation of dynein–microtubule interactions rather than through effects on ciliogenesis per se (Fig. 4B).

Complementary work identified CCP5 as the principal tubulin deglutamylase regulating axonemal glutamylation levels in zebrafish motile cilia [82]. Loss of Ccp5 caused axonemal hyperglutamylation, which impaired ciliary motility and produced ciliopathy-like phenotypes, while paradoxically promoting axoneme assembly in multiciliated cells. Notably, increased glutamylation resulting from CCP5 depletion partially rescued ciliogenesis defects in IFT-deficient backgrounds, demonstrating that tubulin glutamylation can act as an intrinsic driver of axoneme assembly while requiring precise tuning for optimal motility [82].

In mammalian airway epithelia, tubulin polyglutamylation has been shown to be essential for directional beating and effective mucociliary clearance. Using a TTLL1 knockout mouse model, Ikegami et al. demonstrated that loss of TTLL1 leads to a profound reduction of α- and β-tubulin glutamylation in tracheal ciliary axonemes without disrupting the canonical 9 + 2 ultrastructure or dynein arm assembly. Despite remaining structurally intact and motile, TTLL1-deficient cilia lost an intrinsic axonemal curvature that normally biases the effective stroke, resulting in more symmetric beating, reduced fluid flow, and impaired mucociliary transport [42].

Collectively, these studies demonstrate that tubulin glutamylation regulates flagellar and motile cilia function by acting as a spatially patterned axonemal modification that fine-tunes dynein–MT interactions. Rather than serving purely structural roles, glutamylation modulates motor activity, waveform, and beating asymmetry through precise control of its localization and glutamate chain length across distinct axonemal substructures [42, 51–53, 80, 82] (Fig. 4B).

### The roles of glutamylation in primary cilia

Glutamylation profoundly impacts ciliary structure and stability. In *C. elegans* amphid sensory neurons, loss of the deglutamylase CCPP-1 (hyperglutamylation) leads to progressive ciliary degeneration, accompanied by abnormal A–B doublet structures with missing B-tubules [94]. Conversely, hypoglutamylation caused by TTLL6 depletion in zebrafish impairs olfactory cilia assembly [80]. This defect of TTLL6 also leads to structural distortion of axonemal A-tubules [64], indicating that both excessive and insufficient glutamylation can compromise ciliary architecture.

During the cell cycle, ciliogenesis is triggered when cells enter G0, at which point the mother centriole converts into a basal body to initiate axoneme elongation through kinesin-2-mediated IFT [95]. Upon re-entry into mitosis, the Aurora A–HDAC6 (Histone deacetylase 6) pathway triggers ciliary disassembly through tubulin deacetylation [96]. Acute removal of ciliary glutamylation delays ciliogenesis by impairing kinesin-2-dependent transport [61] (Fig. 4B). However, once cilia are formed, hypoglutamylation does not strongly affect maintenance of mature cilia but instead accelerates ciliary disassembly [60, 61]. These findings suggest that glutamylation is important for assembly and disassembly, but plays minimal roles in maintaining mature cilia.

Beyond structural regulation, glutamylation also fine-tunes IFT velocity and sensory signaling within primary cilia. For example, glutamylation level dynamically responds to environmental stimuli. In *C. elegans*, starvation activates the p38 MAPK pathway, which phosphorylates TTLL-4 and accelerates anterograde IFT [40]. Glutamylation is also essential for Hedgehog signaling. Deglutamylation blocks the entry of Smoothened and GLI3 into cilia, whereas hyperglutamylation caused by CCP5 loss restores pathway activity [60, 61, 97]. Similarly, balanced glutamylation is required for proper localization of the mechanosensory ion channel PKD2 (Polycystic kidney disease 2), highlighting the importance of this modification for maintaining the signaling competence of primary cilia [60, 97] (Fig. 4B).

### The roles of tubulin glutamylation in centrosomes and basal bodies

The first insights into centriolar glutamylation came from Michel Bornens’s group, who showed that injection of the GT335 antibody into HeLa cells caused centriole disappearance within 12 h, accompanied by loss of the pericentriolar matrix (PCM) [98]. Further work demonstrated that fragmentation occurs during G2 as a result of Eg5- and dynein-dependent spindle forces [99]. A recent study indicates that acute removal of glutamylation does not disrupt the core centriole structure [42]**,** it reduces the ability of the centrosome to withstand mitotic tension, rendering it susceptible to post-mitotic damage. The PCM itself functions as a mechanical scaffold that stabilizes centrosomal architecture [65, 100]. Glutamylation promotes PCM recruitment via electrostatic interactions [43, 101] (Fig. 4B), suggesting that this modification reinforces centrosomal integrity indirectly by enhancing PCM anchorage rather than by directly stabilizing centrioles.

Glutamylation also extends beyond structural roles to regulate MT nucleation and signaling processes. At centrosomes, glutamylation anchors the NEDD1 (Neural precursor cell-expressed, developmentally downregulated protein 1)/CEP192/γ-tubulin complex through electrostatic interactions (Fig. 4B), thereby facilitating MT regrowth and EB1 (End-binding protein 1) comet formation [43]. Hypoglutamylation impairs this recruitment and consequently leads to defective MT nucleation. In addition, glutamylation also regulates centriolar satellite trafficking—loss of glutamylation causes accumulation of PCM1 and defective delivery of ciliogenesis factors such as Talpid3, thereby delaying ciliary growth [43] (Fig. 4B).

Taken together, although glutamylation negatively regulates purified MT dynamics in vitro, it appears to play relatively minimal roles in structural integrity of its substrates (e.g. centrioles and ciliary axoneme). Instead, glutamylation serves primarily as a molecular platform for recruiting signaling molecules and motors, thereby ensuring proper cellular signaling and functions (Fig. 4B).

### Correlation between axonemal tubulin glutamylation and motile cilia dysfunction in human diseases

Genetic and biochemical studies in unicellular organisms and vertebrates converge on the concept that tubulin glutamylation is dispensable for axonemal assembly but essential for efficient force generation and waveform regulation [53, 80, 102]. Loss of glutamylation selectively impairs dynein-driven bending while leaving the gross axonemal architecture intact, indicating that this modification fine-tunes dynein–MT interactions rather than ciliogenesis itself [52].

Motile cilia of the respiratory tract rely on high-amplitude and coordinated beating to drive mucus transport. Based on animal studies demonstrating reduced beat amplitude and altered waveform under glutamylation-deficient conditions [53, 80], diminished tubulin glutamylation in humans is predicted to weaken mucociliary clearance without eliminating cilia. Clinically, this phenotype resembles forms of primary ciliary dyskinesia (PCD) characterized by preserved axonemal ultrastructure but defective motility [103, 104]. These findings support the idea that impaired regulation of axonemal glutamylation can produce PCD-like functional defects even in the absence of obvious structural abnormalities (Fig. 4B) (Table 1).

**Table 1:** Diseases associated with dysregulated tubulin glutamylation

Types	Associated diseases	Phenotype/clinical manifestation	Glutamylation-related mechanism	Refs
Flagella/Motile cilia	Primary ciliary dyskinesia–like respiratory disorder	Chronic respiratory infections; impaired mucociliary clearance; defective ciliary beating	Reduced axonemal glutamylation weakens dynein-driven sliding between outer doublet microtubules, decreasing ciliary beat amplitude and altering waveform	53, 80, 102
	Airway ciliary dysfunction (TTLL1 knockout)	Reduced ciliary beat asymmetry; impaired mucociliary transport	Loss of axonemal polyglutamylation alters intrinsic curvature of the axoneme and compromises directional beating	42
	Asthenozoospermia (TTLL5 mutation)	Reduced sperm motility despite normal flagellar morphology	Impaired glutamylation of flagellar microtubules weakens dynein-driven motility	107
	Asthenozoospermia (TTLL9 knockout)	Reduced sperm motility	Loss of TTLL9 decreases sperm flagellar polyglutamylation, impairing axonemal motility	81
Primary cilia	Joubert syndrome (CEP41)	Cerebellar malformation; defective ciliary signaling	Loss of CEP41 prevents TTLL6 trafficking into cilia, leading to reduced axonemal glutamylation and structural defects in ciliary microtubules	64
	Joubert syndrome (ARL13B pathway)	Unstable cilia; defective Hedgehog signaling	ARL13B–FIP5–RAB11 pathway defects impair TTLL5/6 entry into cilia, causing axonemal hypoglutamylation and mislocalization of PKD2 and GLI3	60
	Joubert syndrome (ARMC9/ TOGARAM1 module)	Short, unstable cilia; abnormal signaling	Mutations reduce axonemal acetylation and polyglutamylation, destabilizing ciliary microtubules	112
	Retinitis pigmentosa (AGBL5 /CCP5 mutation)	Progressive photoreceptor degeneration	Loss of CCP5 causes hyperglutamylation of axonemal microtubules and disrupts photoreceptor ciliary stability	116–118
	Sensory cilia degeneration (CCPP-1 loss)	Progressive degeneration of amphid sensory cilia	Hyperglutamylation destabilizes axonemal doublets and causes B-tubule defects	94
	Olfactory cilia defects (TTLL6 depletion)	Impaired cilia assembly; axonemal distortion	Hypoglutamylation disrupts olfactory cilia formation and microtubule stability	80
	Ciliopathies (acute axonemal deglutamylation)	Impaired intraflagellar transport and defective Hedgehog signaling	Rapid depletion of axonemal glutamylation disrupts kinesin-2–dependent anterograde IFT and prevents proper ciliary accumulation of Hedgehog pathway components	61
Centrosomes and mitotic spindles	Centrosome dysfunction and ciliopathies (acute centrosomal deglutamylation)	Defective microtubule nucleation; impaired ciliogenesis; mitotic defects	Centrosomal glutamylation recruits the NEDD1/CEP192/γ-tubulin complex through electrostatic interactions to promote microtubule nucleation. Hypoglutamylation disrupts centriolar satellite trafficking and reduces Talpid3 levels, thereby impairing ciliogenesis and spindle assembly	43
	Genome instability (TTLL11 loss)	Lagging chromosomes; micronuclei; aneuploidy	Reduced spindle polyglutamylation disrupts chromosome segregation	119
	Cancer progression (TTLL11 downregulation)	Increased chromosomal instability	Reduced spindle glutamylation promotes aneuploidy	119
	Cancer progression (TTLL4 / TTLL6 overexpression)	Enhanced proliferation; chromatin remodeling	Excess glutamylation promotes tumor-associated cell-cycle progression	120,121
Others	Purkinje cell degeneration (CCP1 deficiency)	Loss of Purkinje neurons; neurodegeneration	Hyperglutamylation disrupts axonal microtubule stability and intracellular transport	25
	Infantile-onset spinocerebellar degeneration (CCP1 mutation)	Cerebellar atrophy; motor neuropathy	Loss of CCP1 leads to excessive neuronal glutamylation	122
	Hereditary spastic paraplegia (Spastin)	Progressive axonal degeneration; spasticity	Excess polyglutamylation inhibits spastin-mediated microtubule severing	87,89
	Axonal degeneration (TTLL11 dysfunction)	Accelerated axonal decay	Loss of TTLL11 disrupts spastin-dependent microtubule remodeling	124
	Tauopathies (e.g., Alzheimer’s disease)	Tau aggregation; neurodegeneration	Long glutamate side chains recruit GSK3 and promote tau hyperphosphorylation	125

Ependymal cilia generate directional cerebrospinal fluid (CSF) flow in vertebrates. Reduced ciliary beating efficiency, as inferred from zebrafish and other model systems with impaired glutamylation [80], is predicted to compromise CSF circulation. Such defects may contribute to hydrocephalus or related neurodevelopmental disorders, consistent with broader links between motile cilia dysfunction and CSF flow abnormalities [105]. Importantly, these observations suggest that insufficient mechanical output of ependymal cilia, rather than ciliary loss, may underlie certain hydrocephalus phenotypes (Fig. 4B) (Table 1).

The requirement of tubulin glutamylation in efficient flagellar beating is conserved across eukaryotes [53, 80, 102]. In sperm, weakened dynein force output without flagellar disassembly provides a plausible mechanism for asthenozoospermia, a form of male infertility characterized by reduced sperm motility despite normal flagellar morphology [105, 106]. Supporting this model, loss-of-function mutations in TTLL5 have been identified in human patients with impaired sperm motility and male infertility [107]. Conversely, pathogenic variants in the deglutamylating enzyme AGTPBP1 (also known as CCP1) have been associated with abnormal spermatogenesis and sperm morphological defects, including flagellar abnormalities, indicating that both insufficient and excessive glutamylation can disrupt flagellar function [108–110].

Collectively, studies across eukaryotic model systems and human genetics demonstrate that dysregulation of tubulin glutamylation primarily causes force-generation defects in motile cilia and flagella, linking post-translational imbalance to human diseases characterized by preserved axonemal structure but compromised mechanical function (Fig. 4B) (Table 1).

### Primary ciliary glutamylation and associated diseases

Lee et al. identified *CEP41* as a gene mutated in patients with Joubert syndrome, a ciliopathy characterized by cerebellar malformations and defective ciliary signaling [64]. CEP41 localizes to the basal body and primary cilium, where it plays a key role in transporting the TTLL6 into the cilium. Loss of CEP41 in human cells, zebrafish, and mice led to impaired delivery of TTLL6, resulting in a dramatic reduction of tubulin glutamylation in cilia. Consequently, although cilia were still present in CEP41-deficient cells, axonemal A-tubules become structurally distorted, sometimes appearing swollen or incomplete [64]. Moreover, Arl13B, a small GTPase localized to the ciliary membrane, and mutations in Arl13B have been identified in patients with Joubert syndrome [111]. He et al. demonstrated that Arl13B regulates axonemal glutamylation by promoting the ciliary entry of TTLL5/6 through the Arl13B–FIP5 (Rab11 family-interacting protein 5) trafficking pathway [60]. When Arl13B or components of its trafficking machinery are deficient, the ciliary axoneme becomes hypoglutamylated, leading to accelerated cilia disassembly and defective ciliary signaling. Consequently, sensory receptors (e.g., Polycystins) and downstream effectors (e.g., GLI3 in the Hedgehog pathway) fail to localize properly within the cilium [60] (Fig. 4B). Latour et al. identified a ciliary protein module in which ARMC9, TOGARAM1, and CCDC66 interact with Joubert syndrome–related proteins CEP104 and CSPP1 [112]. Using protein–protein interaction mapping and patient-derived models, they showed that mutations in ARMC9 or TOGARAM1 lead to reduced tubulin acetylation and polyglutamylation on ciliary axonemes. Loss of these PTMs compromises MT stability, resulting in shortened and unstable cilia accompanied by defective ciliary signaling [112]. Consequently, disruption of this module impairs ciliary structure and function, leading to the neurological and developmental abnormalities characteristic of Joubert syndrome. These works established mechanistic links between axonemal glutamylation and human ciliopathies (Table 1), demonstrating that defects of ciliary glutamylation can contribute to the pathogenesis of Joubert syndrome.

In photoreceptor cells, the outer segment represents a highly specialized primary cilium, and genetic perturbations of glutamylation-regulating enzymes have revealed the importance of this post-translational modification for retinal integrity. In mouse models, loss of the deglutamylase CCP1 results in pathological hyperglutamylation and leads to progressive photoreceptor degeneration [25, 113, 114]. Importantly, genetic suppression of excessive glutamylation rescues neurodegenerative and retinal phenotypes, demonstrating that glutamylation imbalance itself is causative rather than a secondary consequence of degeneration [28, 115]. Consistent with these findings, mutations in AGBL5, encoding the deglutamylase CCP5, have been identified in patients with retinitis pigmentosa, providing direct clinical evidence that disruption of glutamylation homeostasis compromises photoreceptor survival [116–118]. Building on this foundation, a recent study has provided mechanistic insight into how glutamylation imbalance disrupts photoreceptor integrity by revealing the nanoscale organization of tubulin PTMs within the photoreceptor cilium. Using ultrastructure expansion microscopy, the authors demonstrate that glutamylation is highly enriched at the connecting cilium, where it forms a distinct sheath-like structure partially external to axonemal microtubules. Loss of CCP1 or CCP5 results in pathological hyperglutamylation that precedes and drives progressive structural disorganization of the outer segment, characterized by destabilization of the distal axoneme, loss of the bulge region required for disc formation, and severe impairment of IFT. Notably, these defects occur despite preservation of the core connecting cilium scaffold and are not phenocopied by loss of tubulin acetylation, establishing aberrant glutamylation as a primary determinant of photoreceptor ciliary architecture and survival [62]. Overall, these studies demonstrate that dysregulated tubulin glutamylation represents a central pathogenic mechanism linking primary cilium dysfunction to photoreceptor degeneration and retinal ciliopathies (Table 1).

### Centrosomal glutamylation and genome stability

Several recent studies demonstrated that centrosomal glutamylation provides an electrostatic interface for anchoring nucleation factors, including NEDD1, CEP192, and γ-tubulin, thereby securing the proper MT nucleation as well as relative signaling transport [43, 101]. Defects of MT-dependent transport in hypoglutamylated centrosomes or basal bodies indirectly impair ciliogenesis [43], highlighting the important roles of centrosomal glutamylation in ciliopathies. Abnormal MT nucleation can also disrupt mitotic spindle formation, thereby prolonging cell division and compromising chromosome segregation fidelity. Moreover, TTLL11, a mitotic glutamylase, specifically catalyzes the elongation of glutamate side chains on mitotic spindle [119]. Loss of TTLL11 in human cells or zebrafish embryos substantially reduces mitotic spindle polyglutamylation and results in chromosome segregation defects-including lagging chromosomes, micronuclei formation, and aneuploidy-which ultimately impair early embryonic development. Importantly, TTLL11 expression has been found to be systematically downregulated across multiple human cancers, correlating inversely with tumor aneuploidy levels. This downregulation appears to be linked to oncogenic transcriptional programs involving CCNE1 (Cyclin E) and CDC25A [119]. These findings suggest that TTLL11-dependent MT polyglutamylation represents a key mechanism for maintaining genome stability, and that its loss may contribute to the chromosomal instability frequently observed in tumor cells. Furthermore, overexpression of TTLL4 or TTLL6 in several tumors, including breast and pancreatic cancer, also enhances chromatin remodeling and cell-cycle progression [120, 121]. Together, these observations underscore the role of centrosomal and spindle-associated glutamylation as a regulatory rheostat that maintains mitotic fidelity and genome stability (Table 1).

### Neuronal glutamylation and neurodegenerative diseases

In neurons, where long-distance transport along MT is critical, glutamylation is particularly abundant and dynamically regulated during development [1]. Loss of homeostasis in this modification leads to profound neurodegeneration. Rogowski et al. first identified CCP1 as an essential regulator for neuronal survival, showing that Purkinje cell degeneration (PCD) mice lacking CCP1 accumulate hyperglutamylated MTs and rapidly lose Purkinje cells, followed by degeneration of olfactory and thalamic neurons [23]. Mutations in human CCP1 cause infantile-onset spinocerebellar degeneration, characterized by cerebellar atrophy and motor neuropathy [122]. Notably, deletion of glutamylases TTLL1 or TTLL4 in *pcd* mice restores normal polyglutamylation levels and rescues neurodegeneration [123] (Table 1).

Spastin, a MT-severing enzymes mutated in hereditary spastic paraplegia, is also directly regulated by glutamylation. Moderate glutamate chain length enhances spastin activity, whereas excessive polyglutamylation inhibits severing [89] (Fig. 4A), leading to impaired axonal remodeling. TTLL11 further modulates spastin function during axon guidance, and loss of TTLL11 exacerbates axonal degeneration [124]. Beyond hereditary disorders, aberrant glutamylation has been implicated in tauopathies such as Alzheimer’s disease. Glutamate chains on α-tubulin isotype TUBA4A recruit the kinase GSK3 (Glycogen synthase kinase 3), promoting tau hyperphosphorylation and aggregation, whereas reduction of TUBA4A glutamylation suppresses tau pathology in mouse models [125].

Collectively, these studies demonstrate that tubulin glutamylation functions as a unifying molecular regulator linking centrosome stability, ciliary signaling, and neuronal maintenance. In neurons, hyperglutamylation destabilizes axonal cytoskeletons and disrupts intracellular transport; in cilia, hypoglutamylation impairs sensory signaling and organelle biogenesis; and in dividing cells, misregulated centrosomal or spindle glutamylation leads to chromosome segregation errors and tumorigenesis. Maintaining the balance between the “writers” (TTLL enzymes) and “erasers” (CCP enzymes) of the glutamylation code is therefore essential for cellular homeostasis. The enzymes governing this modification—by modulating MT charge, rigidity, and binding affinity—represent promising therapeutic targets for neurodegenerative diseases, ciliopathies, and cancers (Table 1).

## Future perspectives

### Live-cell biosensors for detecting glutamylated MTs

The study of tubulin glutamylation has entered a new era with the advent of advanced molecular and imaging technologies that enable unprecedented spatiotemporal resolution. Currently, live-cell imaging is emerging as a powerful approach to investigate the dynamic regulation of tubulin PTMs and overcome the limitations of traditional methods such as antibody-based immunostaining. Kesarwani et al. developed an analogous biosensor for tyrosinated α-tubulin, enabling real-time observation of MT depolymerization events in living cells under drug treatments [126]. The development of this genetically encoded nanobody-based probe not only demonstrates the feasibility of real-time monitoring in living cells, but also paves the way for uncovering the roles of various PTMs using finely designed biosensors [22]. Several MAPs that have been shown to interact with glutamylated MTs could potentially serve as candidate recognition modules for glutamylated MT biosensors. For instance, MAP1A preferentially binds MT bearing long glutamate chains (~ 7 glutamates), while MAP1B, MAP2, and Tau bind preferentially to moderately glutamylated MTs (1—3 glutamate residues) [127] (Fig. 4A). In addition, the MT-severing enzymes spastin and katanin also associate with glutamylated MTs during severing reactions (Fig. 4A). Several endoplasmic reticulum-associated proteins display affinity for glutamylated MTs as well. Kinectin preferentially interacts with polyglutamylated MTs containing long glutamate chains, whereas p180 shows broader binding preference, interacting with both glutamylated and polyglutamylated MTs regardless of chain length [128]. Furthermore, several centrosomal proteins including CSAP (Cilia and spindle-associated protein) [129], NEDD1/CEP192/γ-tubulin complex also physically interact with centrosomal glutamylated MTs [43]. Recent studies also showed KIF2C, a kinesin-13 family motor protein, preferentially binds to polyglutamylated MTs [130]. Mahalingan et al. further demonstrated that TTLL6 recognizes glutamylated MTs through a specific interaction pocket that senses both the position and the length of glutamate side chains on the CTTs of α-tubulin [131]. Although many of these proteins display affinity for mono- or polyglutamylated microtubules, they also possess additional cellular functions. Therefore, a deeper understanding of the molecular basis of these interactions will be essential for engineering biosensors that selectively detect glutamylation while minimally perturbing endogenous cellular processes. Alternatively, unbiased screening of various MAPs on purified MTs with defined PTMs could reveal novel binding with improved specificity of glutamylation [132]. Together, these advances represent a critical step toward dynamically decoding the “tubulin modification language” in living cells.

### Pharmaceutical modulation of tubulin glutamylation

Current approaches for manipulating tubulin glutamylation mainly rely on genetic approaches, such as modifying the expression of TTLL or CCP enzymes. The development of pharmacological modulators or inhibitors of tubulin glutamylation would greatly facilitate experimental and therapeutic applications across diverse physiological and pathological contexts. Numerous pharmacological inhibitors have already been developed to regulate tubulin acetylation. Acetylation of α-tubulin at Lys40 is maintained by the acetyltransferase αTAT1 and reversed by the deacetylases HDAC6 and SIRT2. Small-molecule inhibitors such as tubacin [133], ACY-1215 (ricolinostat) [134], and AGK2 [135] inhibit deacetylase activity, thereby increasing tubulin acetylation levels. Elevated acetylation stabilizes MTs and enhances cellular resilience to mechanical stress [136, 137]. Clinically, HDAC6 inhibitors are under investigation for the treatment of neurodegenerative diseases and cancers, where restoration of MT acetylation can improve axonal transport or sensitizes tumor cells to chemotherapy [138, 139]. Compared with acetylation, pharmacological modulation of tubulin glutamylation remains largely unexplored. Nevertheless, a recent study identified a small-molecule compound, LDC10, that suppresses TTLL-mediated tubulin glutamylation [140]. Further efforts are needed to develop specific inhibitors or activators targeting tubulin glutamylation machinery, including TTLL and CCP enzymes. The discovery and optimization of such compounds would significantly accelerate research on the biological functions of this PTM and may provide promising therapeutic strategies for diseases associated with dysregulated MT glutamylation.

## Conclusion

Tubulin glutamylation acts as a central regulatory hub that coordinates the ultrastructural and functional diversity of MTs across flagella, motile cilia, primary cilia, centrosomes, and neurons. Rather than functioning as a simple on–off switch, glutamylation operates as a quantitative molecular rheostat, in which the position and length of glutamate side chains, written by TTLL enzymes and erased by CCPs, fine-tune MT dynamics, motor behavior, and the recruitment of signaling complexes. Disruption of this finely balanced tubulin code leads to a broad spectrum of human diseases, including ciliopathies, retinal degeneration, neurodevelopmental and neurodegenerative disorders, and cancers, highlighting the critical physiological importance of glutamylation-mediated MT regulation. Moving forward, the integration of biochemical reconstitution approaches, live-cell PTM biosensors, and spatiotemporal manipulation tools such as STRIP, together with the development of selective small-molecule modulators of glutamylation, will be essential for mapping this modification with high temporal and spatial precision and for establishing causal links between specific glutamylation patterns and defined cellular outputs. Deciphering how the glutamylation landscape is dynamically established and remodeled across distinct cell types and disease states will not only deepen our understanding of the tubulin code but also open new therapeutic opportunities for targeting MT-based pathologies.

## Data Availability

All materials are available from the corresponding author.

## Abbreviations

A-tubule, B-tubule, C-tubule: Subfibers of the microtubule triplet within centrioles
AGBL5: ATP/GTP-binding protein–like 5 (also known as CCP5)
CCP: Cytosolic carboxypeptidase
CEP41: Centrosomal protein 41
CTT: C-terminal tail of tubulin
CSAP: Cilia- and spindle-associated protein
EB1: End-binding protein 1
FIP5: Rab11 family–interacting protein 5
GSK3: Glycogen synthase kinase 3
HDAC6: Histone deacetylase 6
IFT: Intraflagellar transport
MAP: Microtubule-associated protein
MAP4m: Microtubule-associated protein 4 (minimal fragment)
MS: Mass spectrometry
MT: Microtubule
NEDD1: Neural precursor cell–expressed, developmentally downregulated protein 1
PCM: Pericentriolar material
PCD: Purkinje cell degeneration
PKD2: Polycystic kidney disease 2 (TRPP2)
PTM: Post-translational modification
RPGRORF15: Retinitis pigmentosa GTPase regulator isoform ORF15
Shh: Sonic Hedgehog
SIM: Structured illumination microscopy
STRIP: SpatioTemporal Rewriting of Intraciliary PTMs
TTLL: Tubulin tyrosine ligase–like enzyme
U-ExM: Ultrastructure expansion microscopy
